# Supplementary material for: Effects of mixing two legume species at seedling stage under different environmental conditions
Source: PeerJ. 2021 Feb 2;9:e10615. doi: 10.7717/peerj.10615 (PMC7863785; doi:10.7717/peerj.10615)
Supplement: Supplemental Information 2 — Plots with the different treatments of the two legume cover crop species: alsike clover (AC) and black medic (BM) was laid out in a randomized complete block design (RCBD) with Factor (1) three diversity treatments (DIV) of AC:BM (100:0, 50:50 and 0:100), Factor (2) represents three seed densities (50%, 100%, and 150% of the recommended seed density). Counting of the germinated seeds was conducted in 2016 (25 days after sowing) and in 2017 (28 DAS) on an area of 0.5 of 24 sections in 2016 (8 rows × 3 blocks) and 48 sections in 2017 (12 rows × 4 blocks). [file peerj-09-10615-s002.pdf]

- **Two legume species**: alsike clover (**AC**) and black medic (**BM**)

Factor 1 (*DIV*): 3 diversity treatments of AC:BM ratio

DIV1: mono (100:0)

DIV2: Mix (50: 50)

DIV3: mono (0:100)

Factor 2 (*Den*): 3 seed densities (of the recommended seed density ;%)

Den1: 50%, **Den2**: 100%, **Den3**: 150%

- 2 years (**y1**: 2016, **y2**: 2017)

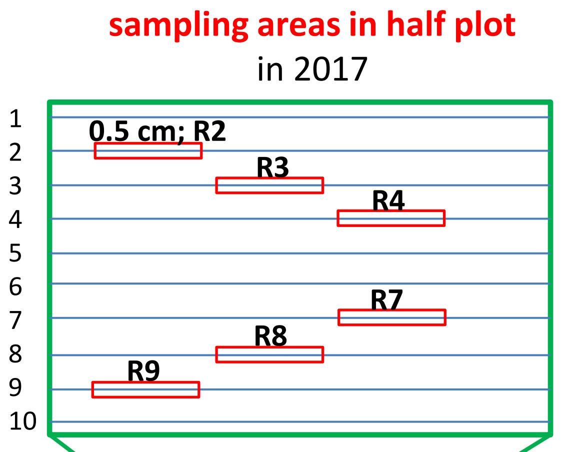

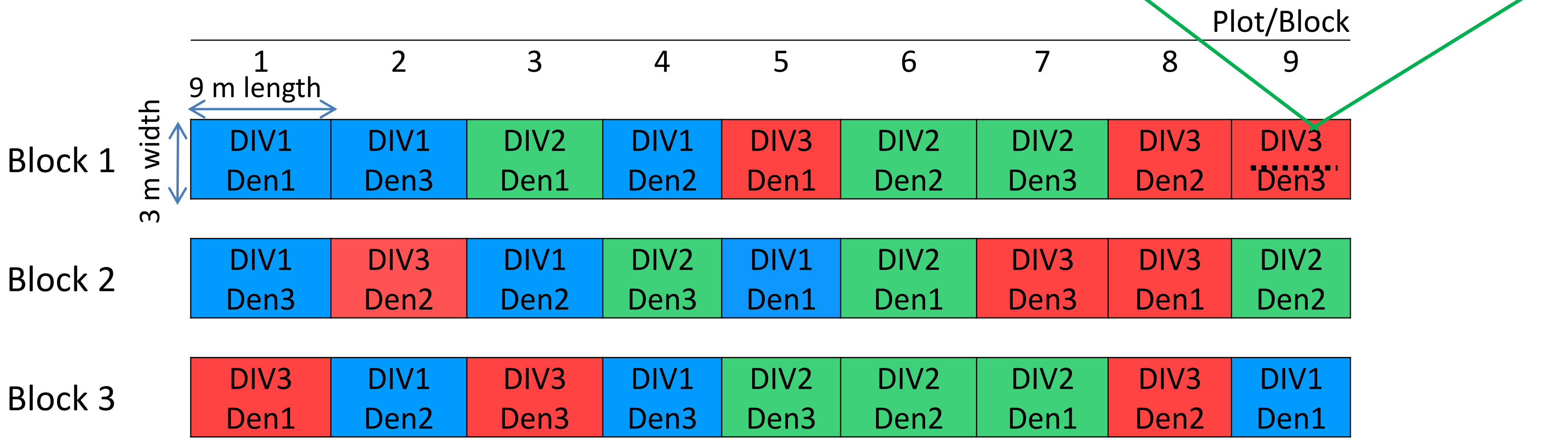

...... The dashed line indicates splitting of each plot into 2 parts; each contains 10 rows.

Number of samplings per density were:

In 2016 = 8 rows /plot x 3 blocks = 24

In 2017 = 12 rows/plot x 4 blocks = 48
