# Supplementary figures and images for: Effects of mixing two legume species at seedling stage under different environmental conditions
Source: PeerJ. 2021 Feb 2;9:e10615. doi: 10.7717/peerj.10615 (PMC7863785; doi:10.7717/peerj.10615)

**Alsike clover; AC**

**28°C**

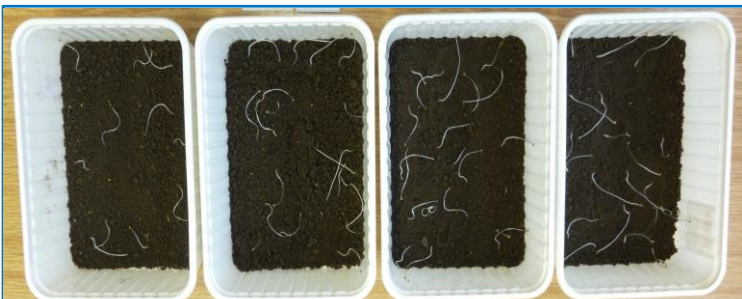

**20°C**

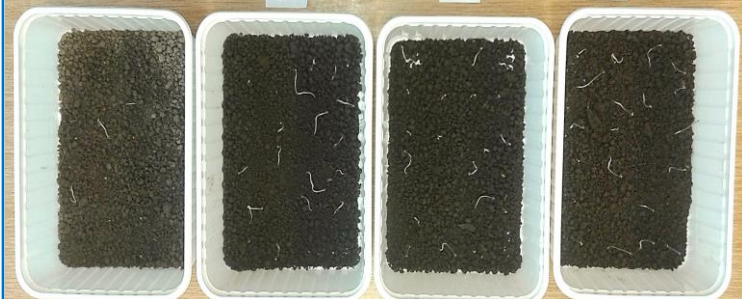

**12°C**

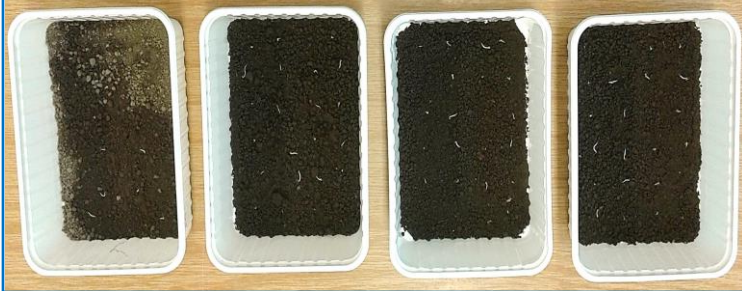

25      50      75      100

**Water holding capacity (%)**

**Black medic; BM**

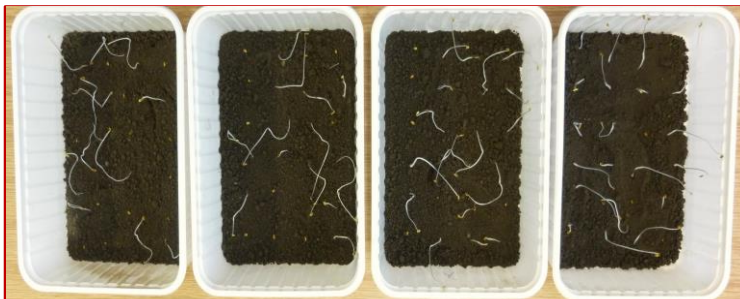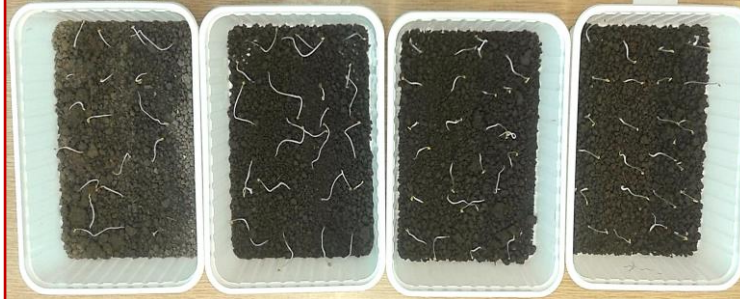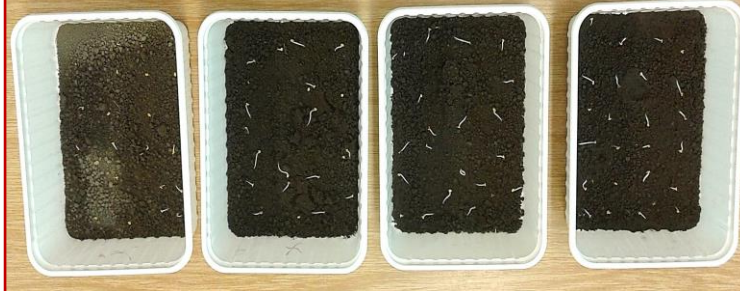

25      50      75      100

**Water holding capacity (%)**

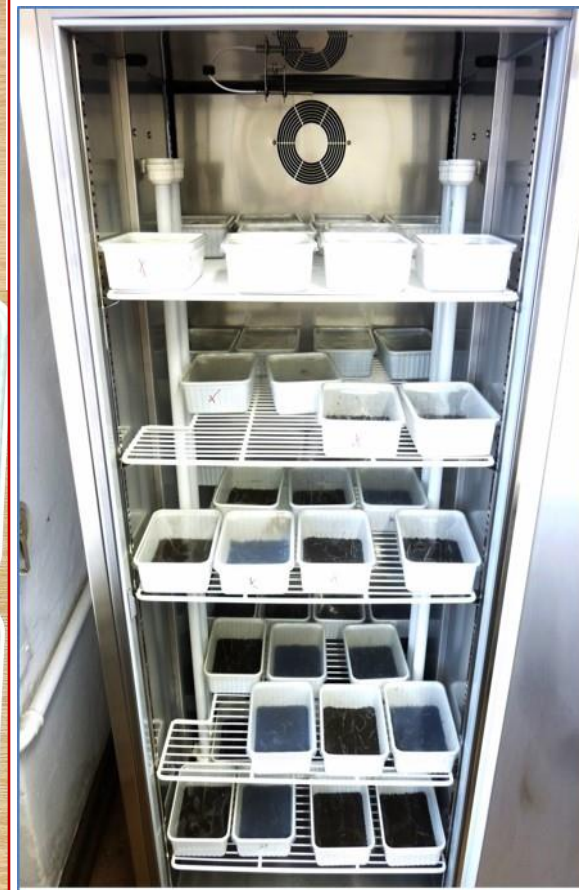

Supplement: Supplemental Information 3 — The seeds are germinated under four drought intensities (100%, 75%, 50%, and 25% WHC) at three temperature levels (12°C, 20°C, and 28°C). The sown seeds are 24 seed box-1. [file peerj-09-10615-s003.pdf]

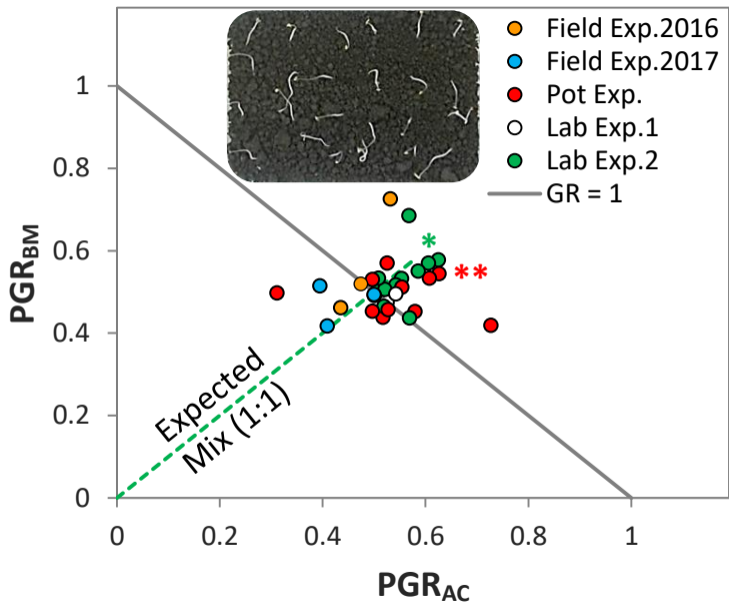

Supplement: Supplemental Information 4 — The different colored circles represent two field experiments (Orange circles; 2016 and blue circles; 2017), pot experiment (red circles), laboratory experiment 1 (white circles), and laboratory experiment 2 (green circles). The solid grey lines correspond to a GR = 1 and the broken green lines correspond to the expected PGR for the mixture. Asterisks above some data points represent a significant increase in GR >1 (P < 0.05) according to Welch’s t-test; ** = P < 0.01, * = P < 0.05. [file peerj-09-10615-s004.pdf]
